# Dapagliflozin Ameliorates Doxorubicin-Induced Chemobrain and Cognitive Abnormalities in Rats: Modulation of AKT/GSK-3β and Wnt/β-Catenin Pathways

**DOI:** 10.1007/s11064-025-04538-0

**Published:** 2025-09-05

**Authors:** Gehad Farouk Abdelhafez, Sylvia A. Boshra, Hagar B. Abo-Zalam, Sara M. Radwan

**Affiliations:** 1 Biochemistry Department, Faculty of Pharmacy, October 6 University, Giza, Egypt; 2 Pharmacology and Toxicology Department, Faculty of Pharmacy, October 6 University, Giza, Egypt; 3 Biochemistry Department, Faculty of Pharmacy, Ain Shams University, Cairo, Egypt

**Keywords:** Doxorubicin, Chemobrain, Dapagliflozin, Cognitive function

## Abstract

Cognitive impairments are frequently observed in cancer survivors who received chemotherapy based on doxorubicin (DOX), attributable to oxidative stress, neuroinflammation, and the apoptotic effect of DOX. Dapagliflozin (DAPA) has gained significant attention attributable to its powerful anti-inflammatory, antioxidant, and anti-apoptotic characteristics. The present investigation seeks to assess the possible neuroprotective properties of DAPA in alleviating neurodegeneration and cognitive dysfunction caused by DOX. Chemobrain was induced by DOX (2 mg/kg, i.p.) once weekly for four weeks. Additionally, rats were treated with DAPA (2 mg/kg, p.o.) for 28 consecutive days. DAPA markedly mitigated behavioral deficits associated with cognitive impairment induced by DOX, as demonstrated by cognitive behavioral tests. Likewise, DAPA reversed histopathological abnormalities. Mechanistically, DAPA alleviated the oxidative stress induced by DOX by reducing tissue levels of NADPH oxidase 4 (NOX4) and malondialdehyde (MDA) while enhancing the activities of superoxide dismutase (SOD) and glutathione (GSH). Additionally, DAPA suppressed neuroinflammation by attenuating the production of interleukin-1β (IL-1β), tumor necrosis factor-alpha (TNF-α), and nuclear factor kappa-B (NF-κB) in brain tissue, as well as the signaling pathways involved in apoptosis, including the Protein Kinase B (AKT)/Glycogen Synthase Kinase-3 beta (GSK-3β) pathway and the Wingless-related integration site (Wnt)/β-catenin pathway. Further evidence of DAPA’s anti-apoptotic effects was attributed to a decrease in the immunohistochemical expression of total caspase-3 and the p65 subunit of NF-κB (NF-κB-p65). DAPA induces neuroprotection against DOX-induced cognitive deterioration by providing antioxidant, anti-inflammatory, and anti-apoptotic effects.

**Graphical Abstract:**

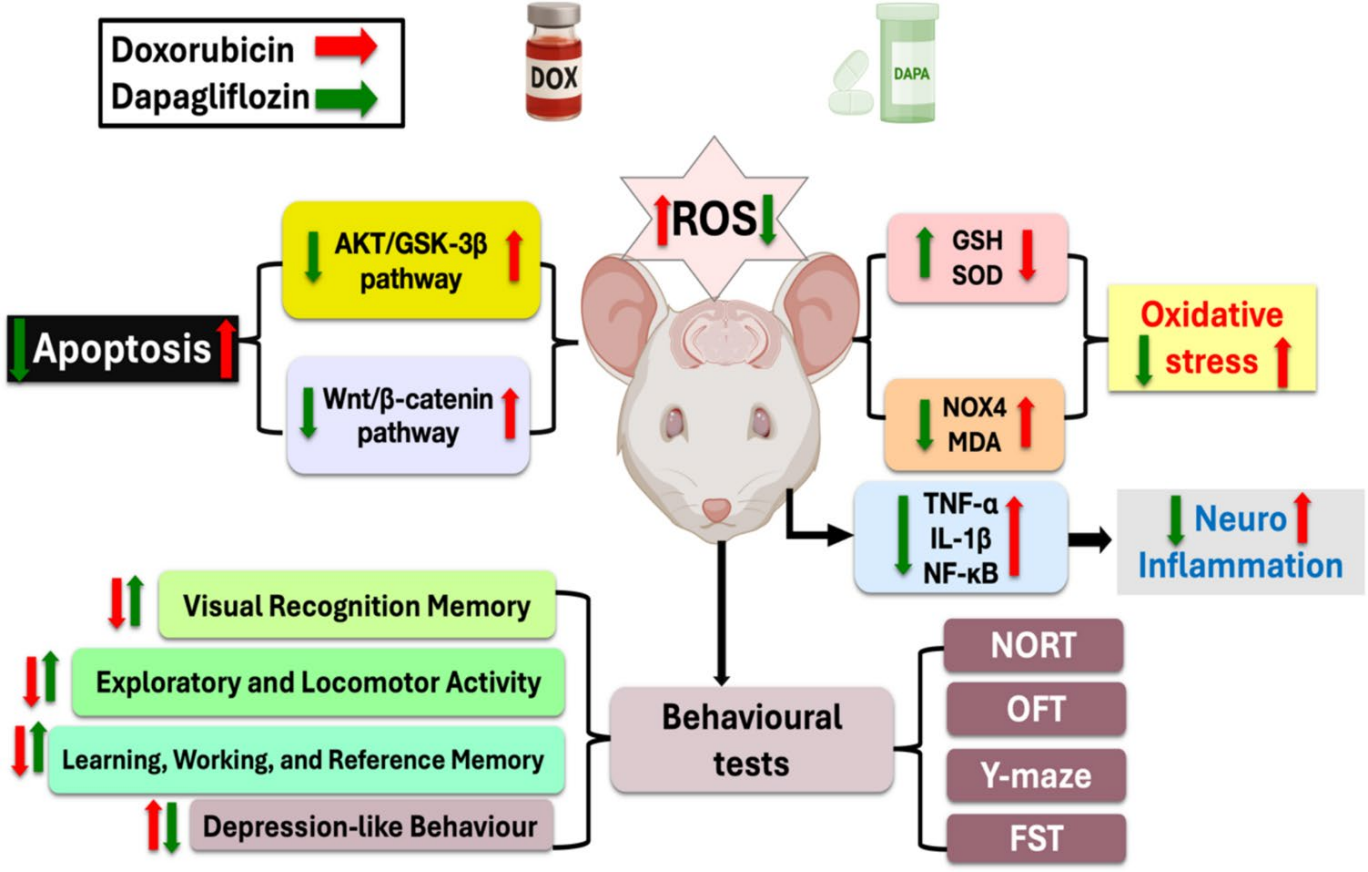

**Supplementary Information:**

The online version contains supplementary material available at 10.1007/s11064-025-04538-0.

## Introduction

The term “chemobrain” refers to the cognitive and mental impairments experienced by cancer patients throughout and following chemotherapy. It is commonly termed “chemobrain” or “chemo-fog.” Chemobrain develops in patients following chemotherapy for various reasons, including neural inflammation, stress from free radical formation, and abnormalities in normal neuronal cell processes caused by metabolic changes. The major mediators of chemobrain are pro-inflammatory cytokines, including tumor necrosis factor (TNF), interleukin-1 (IL-1), and IL-6. Continuous chemotherapy leads to a significant increase in oxidative stress, which in turn decreases neurogenesis and gliogenesis [1]. Memory impairment, loss of concentration, speech and psychomotor slowdown, problems with attention and learning coordination, and executive function disruption are all signs of chemobrain. The symptoms may be temporary, but they are frequently long-lasting, significantly impacting functionality and quality of life. Presently, there is no therapeutic plan for chemobrain-related cognitive impairment, while numerous pharmacotherapies are being investigated [2]. Doxorubicin (DOX) is a broadly prescribed anthracycline-based antineoplastic drug, approved by the FDA for its proven efficacy in treating various types of tumors. DOX has cytotoxic consequences via multiple mechanisms, encompassing DNA intercalation and topoisomerase II inhibition [3]. Furthermore, the overproduction of reactive oxygen species (ROS) caused by DOX’s redox cycle is a major factor in cell death via the aforementioned pathways. DOX chemotherapy results in multi-organ toxicities, including nephrotoxicity, hepatotoxicity, hematological toxicity, dose-limiting fatal cardiotoxicity, and brain neurotoxicity, which is interpreted as a decline in brain function [3]. For cancer survivors, one of the most difficult issues undergoing treatment based on DOX is cognitive decline. DOX induces oxidative stress via decreasing glutathione (GSH) and superoxide dismutase (SOD) and elevating NADPH oxidase 4 (NOX 4) and malondialdehyde (MDA) levels [4, 5]. Neuroinflammation is believed to be primarily responsible for chemobrain caused by DOX. Intriguingly, the neuroinflammatory response to DOX results in the overexpression of the nuclear factor kappa-B (NF-κB) gene and enhanced release of proinflammatory mediators, including IL-1β and TNF-α [6], alongside the signaling pathways of Protein Kinase B (AKT)/Glycogen Synthase Kinase-3 beta (GSK-3β) and the Wingless-related integration site (Wnt)/β-catenin [7, 8].

The European Medicines Agency (EMA) has authorized dapagliflozin (DAPA), the first novel sodium-glucose co-transporter-2 (SGLT-2) inhibitor, to manage type 2 diabetes by blocking SGLT2. DAPA reduces blood glucose levels and increases urine glucose excretion by blocking the kidney’s capacity to reabsorb filtered glucose [9]. In addition to its anti-diabetic actions, recent experimental evidence supports the neuroprotective role of DAPA in multiple models of neurological disorders [10, 11]. This may be due to its capacity to counteract the negative effects of ROS that are generated in reaction to neurotoxic stimuli [12].

It has been documented that DAPA’s encouraging potential as a neuroprotective agent is signified through inhibition of ROS-induced neuronal death, suppression of AKT/GSK-3β and Wnt/β-catenin pathways, and reduction of neuroinflammation via reducing NF-κB activation and TNF-α levels [13]. The aim of this study was to explore the possible therapeutic effectiveness of dapagliflozin on cognitive decline triggered by doxorubicin in rats utilizing behavioral, histological, immunohistochemical, and biochemical assessments.

## Materials and Methods

### Animals

Forty male albino Wistar rats were sourced from the Animal House Colony of the National Research Centre (NRC) in Giza, Egypt, weighing between 150 and 200 g. The animals were given a week to acclimate before the study began. The animals were placed in appropriate cages (5 rats/cage) in an air-conditioned room with alternating 12-h day/night cycles at 23 ± 2 °C. The animals received an unlimited water supply and standardized food pellets. Throughout the experiment, all attempts were made for the animals to reduce the degree of pain or suffering that may have been caused. The study was ethically approved by the Research Ethics Committee (REC) of the Faculty of Pharmacy, Ain Shams University, Cairo, Egypt (ACUC-FP-ASU REC#180, 2023), ensuring compliance with institutional and international standards.

### Chemicals and Drugs

Doxorubicin (Doxorubicin®) was purchased from Ebewepharma (Unterach, Austria). In addition, DAPA, or dapagliflozin (Forxiga®), was purchased from AstraZeneca (Giza, Egypt). All highest purity grade chemicals and solvents were stored at temperatures between 2 and 8 °C.

### Experimental Protocol

**Fig. 1:**
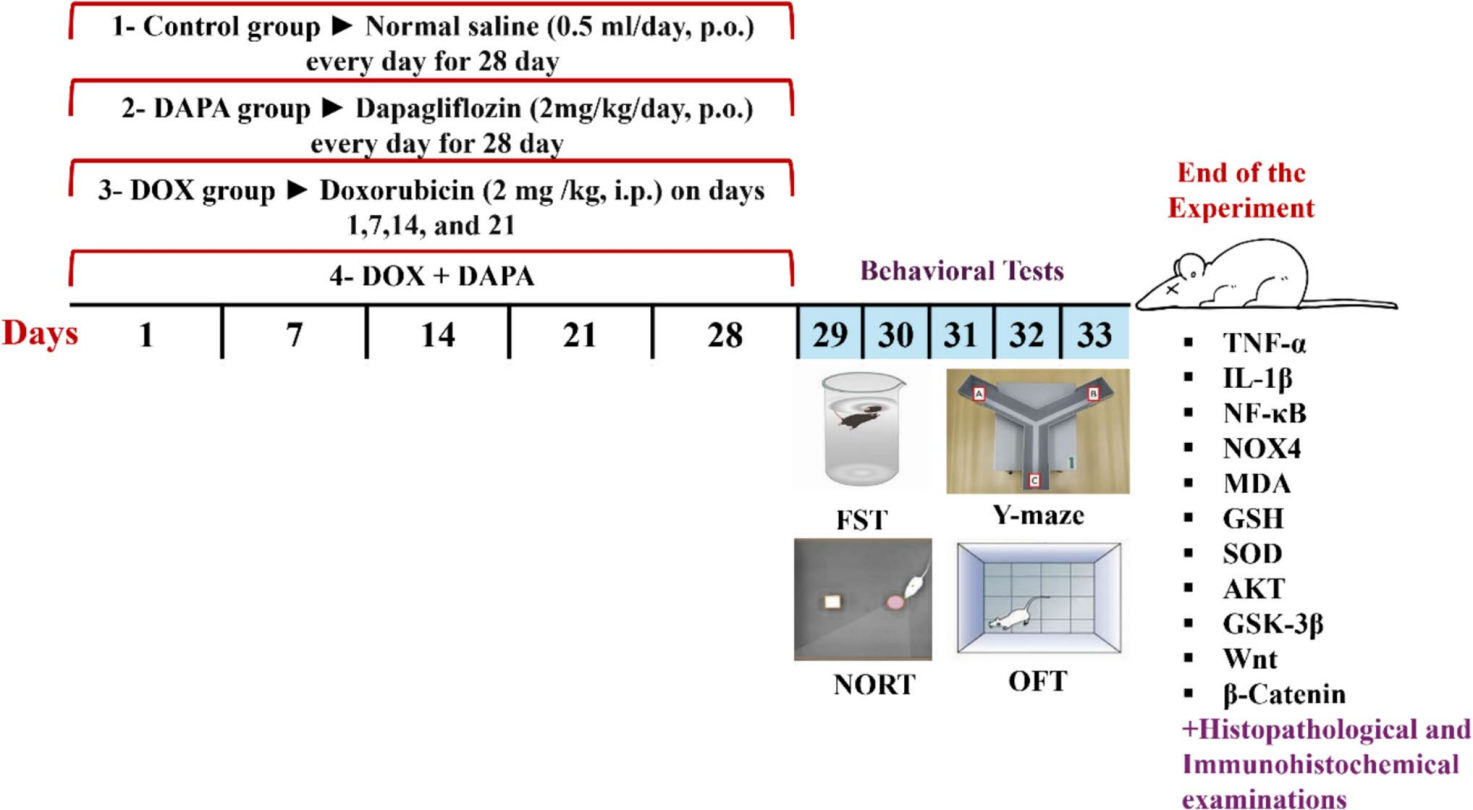
Experimental protocol**.** Animals were given DOX (2 mg/kg, i.p.) once every week for 28 days (on the experiment’s days 1, 7, 14, and 21). DAPA in a dose of 2 mg/kg, p.o., was given daily for 28 days beginning on the first day of the study schedule, and behavioral tests were carried out from day 29 to 33; then, on the next day, animals were euthanized. Brain tissues were separated for biochemical, histopathological, and immunohistochemical analyses. Where *AKT* protein kinase B, *DAPA* Dapagliflozin, *DOX* Doxorubicin, *FST* Forced Swimming test, *GSH* Reduced glutathione, *GSK-3β* Glycogen synthase kinase-3beta, *i.p* Intraperitoneal, *IL-1β* Interleukin-1β, *MDA* Malondialdehyde, *NF-κB* Nuclear factor kappa-B, *NORT* Novel Object Recognition test, *NOX4* NADPH oxidase 4, *OFT* Open Field test, *p.o* Orally, *SOD1* Superoxide dismutase 1, *TNF-α* Tumor necrosis factor-alpha, *Wnt* Wingless-related integration site, and *β-Catenin* beta-Catenin

All rats were distributed randomly into four groups (10/group) (Fig. 1). Group 1: Injected with normal saline intraperitoneally for 28 days and considered as the normal control group. Group 2 (DAPA group): Animals were administered dapagliflozin (2 mg/kg/day, p.o.) starting on the first day of the experiment for 28 days [14, 15]. Group 3: Animals were given doxorubicin (DOX; 2 mg/kg, i.p.) once every week (on experiment days 1, 7, 14, and 21) [16, 17]. Group 4 (DOX + DAPA): Animals were administered doxorubicin (2 mg/kg, i.p.) once every week for 28 days (on experiment days 1, 7, 14, and 21) with dapagliflozin (2 mg/kg/day, p.o.) starting on the 1st day of the experiment for 28 consecutive days. DOX and DAPA were freshly prepared every day and dissolved in a saline solution.

Cognitive behavioral tests were conducted 24 h following the last DAPA dose. The Novel Object Recognition test (NORT) was applied across three days, on experiment days 29, 30, and 31. The Open Field test (OFT) was carried out on experiment day 30, and the Y-maze test was applied on experiment day 31. Days 32 and 33 of the experiment were implemented for the Forced Swimming Test (FST).

After the behavioral assessments on day 34, thiopental sodium (50 mg/kg) was given to euthanize the animals in each group, and they were killed via cervical dislocation. After that, brain tissues were quickly separated, collected, and washed with ice-cold normal saline. Brain samples were split and isolated into two distinct sets. Tissue samples from the first set (n = 6) were kept for enzyme-linked immunosorbent assay (ELISA) and Western blot analyses. For the second set, 10% formalin was used to preserve the tissue samples (n = 3) for histopathological and immunohistochemical analyses. Then, all animals’ cadavers were frozen till incineration.

### Cognitive Behavioral Tests

#### Novel Object Recognition Test (NORT)

NORT was completed over three days (on experiment days 29 to 31): habituation day (day 29), training day (day 30), and testing day (day 31). Rats were allowed to investigate two identical objects during the training day. On the test day, one of the training objects was substituted with a new object. Rats exhibit an inherent preference for novel stimuli; thus, when presented with a familiar object, they allocate the majority of their time to the novel object [18]. The ANY-maze video tracking system (Version 7.36, Wood Dale, USA) was used to analyze the animal’s behavior by tracking the number of entries to the familiar object, the number of entries to the novel object, the familiar object exploration time, and the novel object exploration time. The NORT assesses visual recognition memory, which enables comparing previously stored information with presented stimuli [19].

#### The Open Field Test (OFT)

OFT was applied on the 30th day of the experiment. It is a widely used indicator of behavior and general activity in an open field over time (5 min) [20]. The animal’s behavioral analysis was conducted using the ANY-maze video tracking system (Version 7.36, Wood Dale, USA) by tracking the distance traveled, line crossing, number of corner zone entries, and mean speed. It is employed in the assessment of rats’ emotionality, general locomotor activity, and exploratory behavior [21].

#### The Y-maze Test

On day 31 of the experiment, the rats’ willingness to explore novel settings was evaluated through the Y-maze test. Usually, rats would rather explore a new maze arm than go back to one they have already explored. Testing takes place in a Y-shaped maze with three opaque arms spaced 120° apart. After an introduction to the maze’s center, the animal was allotted 8 min to freely explore the three arms. This test is used for the evaluation of novel medications for their impact on cognition [22]. The behavioral analysis was carried out using the ANY-maze video tracking system (Version 7.36, Wood Dale, USA) by tracking the total distance, number of zone A entries, number of zone B entries, and number of zone C entries. Rats’ spatial reference memory can be evaluated well with the Y-maze test. It investigates working memory, reference memory, and discriminative learning [23].

#### Forced Swimming Test (FST)

The FST is a behavioral test utilized to evaluate behavioral despair and “depressive-like” states in rodents [24]. On experiment days 32 and 33, the rats were kept for 5 min at a height of 20 cm in a plastic cylinder that measured 30 cm in height and 20 cm in width, containing water at 24 ± 1 °C. The animals were exposed to an identical cylinder for 5 min in the second trial, which was carried out 24 h after the first trial [24]. The animal’s behavioral analysis was performed manually by keeping track of the latency time, swimming time, immobility time, and struggling time.

### Histopathological Examination

After being stored in 10% neutral buffer formalin, the brain tissues were carefully dissected, cleaned with water, dehydrated in increasing ethyl alcohol grades, cleared with xylene, and then embedded in paraffin. Thin Sections (4–6 µm) were processed and stained with hematoxylin & eosin [25].

### Immunohistochemistry (IHC) Examination

Paraffin slices were fixed onto positively charged slides. Immunohistochemical staining was conducted utilizing the Avidin–Biotin-Peroxidase Complex (ABC) method. Two polyclonal antibodies were used: rabbit NF-κB (p65) (1:100; Cat#E-EL-R0674, Elabscience, USA) and rabbit total caspase-3 (1:100; Cat#E-AB-63602, Elabscience, USA) (Supplementary File). After incubation with the primary antibodies, ABC reagents from the Vectastain kit (Vectastain ABC-HRP kit, Vector Labs, Newark, USA) were applied per the manufacturer’s instructions. Diaminobenzidine (DAB) (DAB; Sigma-Aldrich, USA) was utilized for visualization of the antigen–antibody complexes, which produced a brown precipitate indicating positive immunoreactivity. Negative controls were included by replacing the primary or secondary antibody with non-immune serum. IHC-stained sections were observed using an Olympus microscope (BX-53, Olympus Corporation, Tokyo, Japan).

### Quantitative Assessment of Biochemical Markers in the Brain Tissue Homogenates by ELISA and Colorimetric Techniques

Using a polytron homogenizer at 4 °C, 10% of the brain tissues were homogenized in 0.05 mol/L of phosphate buffer (pH 7). The homogenate was centrifuged for 20 min at 10,000 rpm to eliminate mitochondria, erythrocytes, nuclei, intact cells, and cell debris. Rat NOX4 ELISA kit (Cat#NBP2-76792, Novus Biological, USA), rat MDA ELISA assay kit (Cat#MBS268427, MyBioSource, San Diego, CA, USA), and SOD1 Rat ELISA kit (Cat#E4584-100, Biovision, Milpitas, CA, USA) were utilized for the determination of oxidative stress biomarkers within the brain samples. Furthermore, rat IL-1β ELISA kit (Cat#E0119Ra, Bioassay Technology Lab, Shanghai, China), rat TNF-α ELISA kit (Cat#438206, Biolegend, Sandiego, CA, USA), and rat NF-κB ELISA kit (Cat#E-EL-R0674, Elabscience, USA) were used for the assessment of the inflammatory markers within the brain samples. A sandwich ELISA format was used, where each kit contained pre-coated antibody plates, biotin-conjugated detection antibodies, enzyme reagents, and chromogenic substrates. All reagents, working standards, controls, and samples were prepared and brought to room temperature before use. A biotin-conjugated detection antibody specific to the collected antigen was then added to bind it. Next, avidin-horseradish peroxidase (HRP) was applied to bind to the biotin, the tetramethylbenzidine (TMB) substrate binds to the HRP enzyme to form a blue color, and sulfuric acid stop solution was used to stop the reaction, producing a yellow color, and the optical density (O.D.) of the well was measured at 450 nm, with corrections at 540 nm or 570 nm using a filter-based multi-mode microplate reader (Stat Fax 2200, Awareness Technologies, Florida, USA). Sample concentrations were calculated using a standard curve by interpolating their optical densities. In addition, GSH was assayed colorimetrically using a reduced glutathione colorimetric assay kit (Cat#K464-100, Biovision, Cairo, Egypt). All procedures followed the kit manufacturer’s instructions.

### Western Blotting (WB)

Following the manufacturer’s instructions, the ReadyPrep™ Protein Extraction Kit (Cat# 163-2086, Bio-Rad, Hercules, CA, USA) was applied to extract the total protein from the brain tissue samples. Protein concentrations in each sample were measured using the Bradford Protein Assay Kit (Cat# SK3041, Bio Basic, Markham, Ontario, Canada) [26]. An equivalent amounts of protein (20 µg) were mixed with the same volume of 2 × Laemmli sample buffer containing 4% sodium dodecyl sulfate (SDS), 10% 2-mercaptoethanol, 20% glycerol, 0.004% bromophenol blue, and 0.125 M Tris–HCl (pH 6.8), then boiled at 95 °C for 5 min to ensure complete denaturation. Sodium dodecyl sulfate–polyacrylamide gel electrophoresis (SDS–PAGE) was used for protein separation by using the TGX Stain-Free™ FastCast™ Acrylamide Kit (Cat# 161-0181, Bio-Rad, CA, USA). Proteins were then transferred to polyvinylidene difluoride (PVDF) membranes using the Trans-Blot® Turbo™ Transfer System (Cat# 1704150, Bio-Rad, CA, USA) at 25 V for 7 min in a transfer buffer composed of 25 mM Tris, 190 mM glycine, and 20% methanol. Membranes were blocked with 3% BSA in TBS-T (20 mM Tris, 150 mM NaCl, 0.1% Tween-20, pH 7.5) for an hour at room temperature. Protein Kinase B (AKT) (1:1000; Cat# 9272, Cell Signaling Technology, MA, USA), Glycogen Synthase Kinase-3 beta (GSK-3β) (1:1000; Cat# 9315, Cell Signaling, Danvers, MA, USA), Wingless-related integration site (Wnt) (1:100; Cat# sc-514531, Santa Cruz Biotechnology, CA, USA), and Beta-Catenin (β-Catenin) (1:200; Cat# sc-7963, Santa Cruz Biotechnology, CA, USA) antibodies were used. After washing 3–5 times (5 min each) with Tris-Buffered Saline with Tween 20 (TBST), membranes were incubated for 1 h at room temperature with Horseradish Peroxidase–conjugated (HRP-conjugated) secondary antibody (goat anti-rabbit IgG-HRP, 1:1000; Novus Biologicals, USA), then washed again in TBST. For signal detection, a chemiluminescent substrate (Cat# 1705060, Bio-Rad, Hercules, CA, USA) was applied by mixing equal volumes of Clarity™ Western Luminol/Enhancer and Peroxidase Solutions. A charge-coupled device (CCD) camera-based imaging system (ChemiDoc™ MP, Cat# 12003154, Bio-Rad, CA, USA) was utilized for visualizing the protein bands. Using image analysis software, band intensities were measured, and as a housekeeping control, each target protein’s expression was normalized against β-actin.

### Statistical Analysis

Data were presented as means ± S.D. Groups comparison was performed by one-way analysis of variance (ANOVA) followed by Tukey’s test. GraphPad Prism (version 5.0, GraphPad Software, Inc., San Diego, USA) was used for all statistical analysis and graphs. P < 0.05 was considered statistically significant.

## Results

### Impact of Dapagliflozin on Novel Object Recognition Test (NORT)

**Fig. 2:**
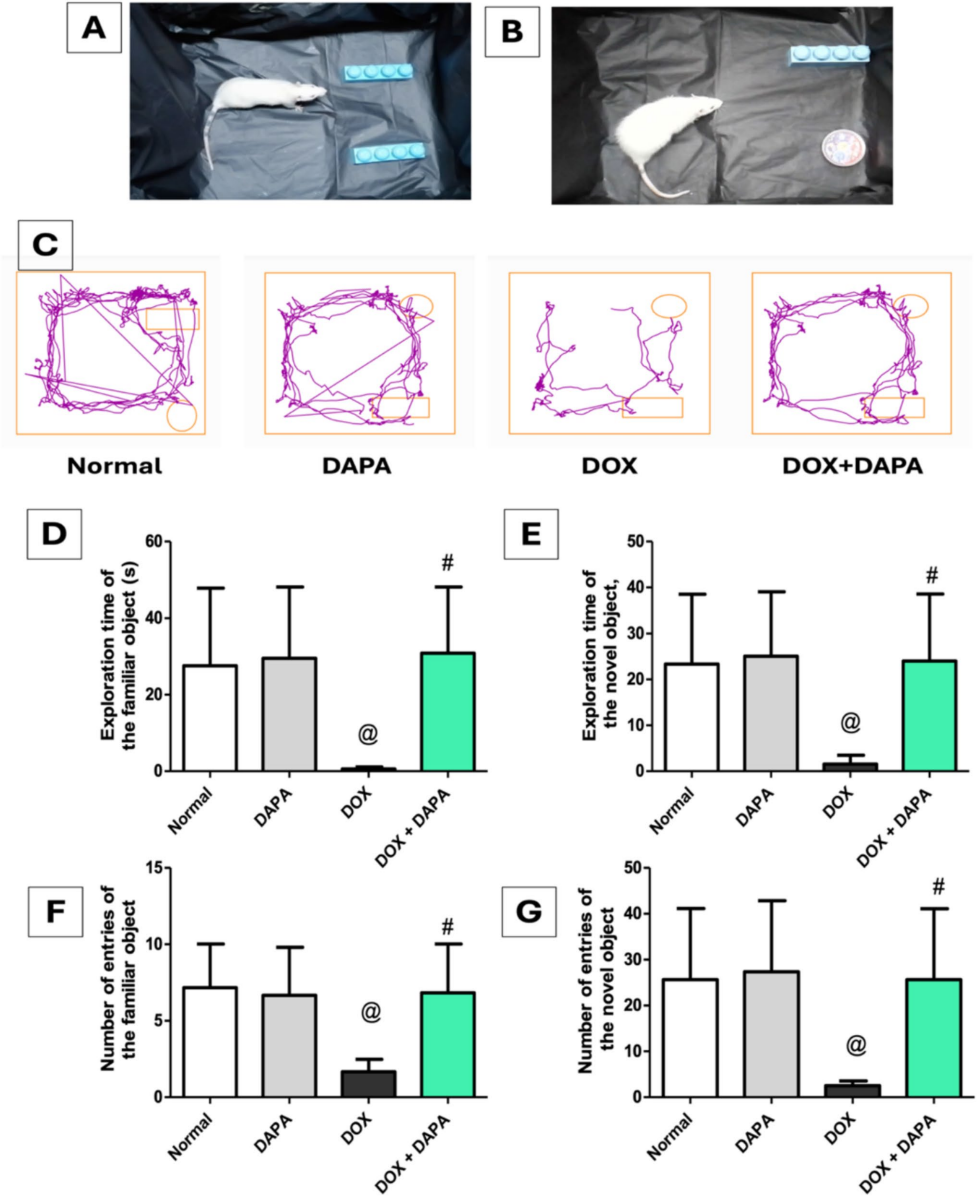
Effect of dapagliflozin on novel object recognition test (NORT) against doxorubicin-induced chemobrain in rats. **A** rats on training day, **B** rats on testing day, **C** track plot of novel object recognition test, **D** exploration time of the familiar object, **E** exploration time of the novel object, **F** number of entries of the familiar object, and **G** number of entries of the novel object. *DAPA* Dapagliflozin, *DOX* Doxorubicin. **@:** vs. the control group & **#:** vs. DOX-intoxicated group, P < 0.05

Following the completion of the NORT (Fig. 2), DOX administration significantly impaired behavioral activity, evidenced by substantial reductions in exploration time for both familiar and novel objects, with decreases of 47.59-fold and 15.08-fold, respectively, as well as reductions in the number of entries to familiar and novel objects by 4.29-fold and 8.37-fold, respectively, relative to the control group. These findings demonstrate that DOX treatment diminishes the preference of rats for the novel object, thereby affecting their discrimination ability and memory sensitivity. In contrast, co-treatment with DAPA showed marked increases in the exploration time of familiar objects and novel objects by 53.22-fold and 15.5-fold, respectively. Furthermore, the number of entries to the familiar object as well as the novel object were also increased by 4.09-fold and 9.2-fold, respectively, in contrast to the DOX group. These results indicate that DAPA effectively restored visual recognition memory and enhanced performance in recognition memory tasks.

### Impact of Dapagliflozin on Open Field Test (OFT)

**Fig. 3:**
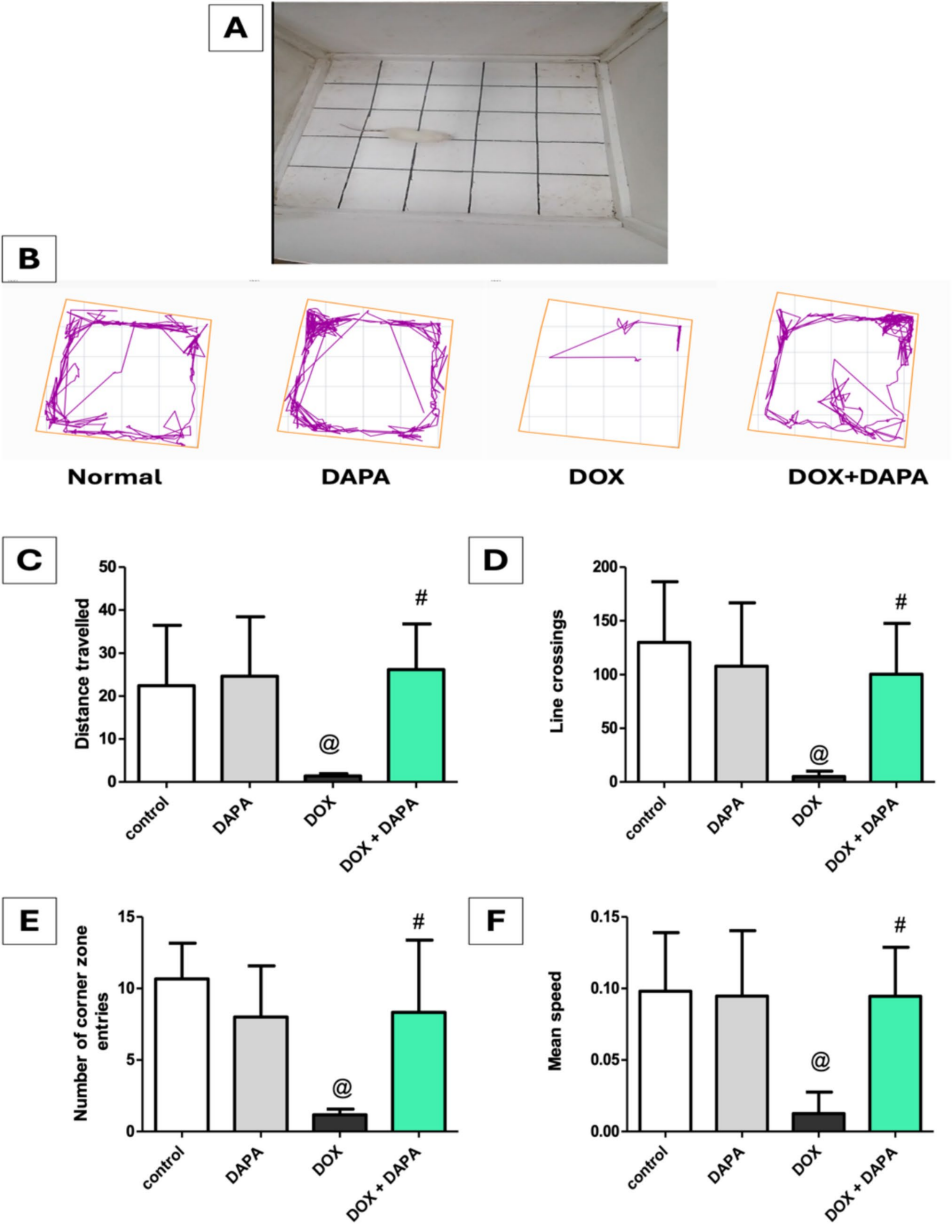
Effect of dapagliflozin on open field test (OFT) against doxorubicin-induced chemobrain in rats. **A** rats on open field test apparatus, **B** track plot of the open field test, **C** distance traveled, **D** line crossing, **E** number of corner zone entries, and **F** mean speed. *DAPA* Dapagliflozin, *DOX* Doxorubicin. **@:** vs. the control group & **#:** vs. DOX-intoxicated group, P < 0.05

The OFT findings **(**Fig. 3**)** indicate that DOX administration reduced the distance traveled and line crossing by 15.92-fold and 26-fold, respectively, while decreasing the number of corner zone entries and mean speed by 9.12-fold and 10-fold, respectively, relative to the control group. Conversely, DAPA co-administration reversed the effects of DOX by increasing distance traveled and line crossing by 18.57-fold and 20.06-fold, while increasing the number of corner zone entries and mean speed by 7.12-fold and 9.0-fold, respectively, as compared to the DOX group. As expected, DAPA-treated rats displayed improved OFT exploratory behavior and general locomotor activity.

### Effect of Dapagliflozin on Y-maze Test

**Fig. 4:**
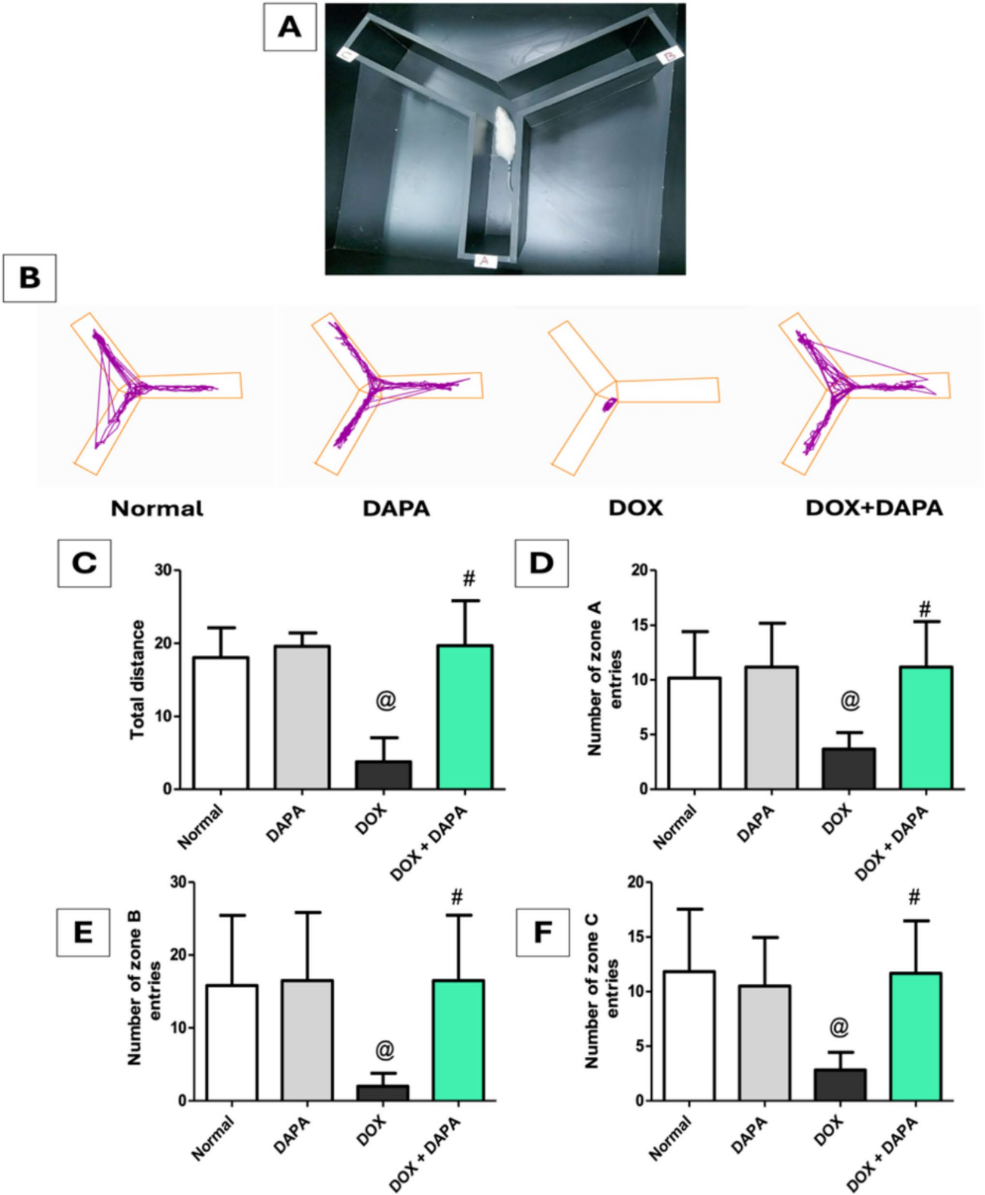
Effect of dapagliflozin on Y maze test against doxorubicin-induced chemobrain in rats. **A** rats on Y-maze test apparatus, **B** track plot of Y-maze test, **C** total distance, **D** number of zone A entries, **E** number of zone B entries, and **F** number of zone C entries. *DAPA* Dapagliflozin, *DOX* Doxorubicin. **@:** vs. the control group & **#:** vs. DOX-intoxicated group, P < 0.05

The outcomes of the Y-maze test demonstrated the detrimental impact of DOX on the animals’ capacity to recall previously visited arms, where the administration of DOX revealed significant reductions in total distance traveled by 4.82-fold, in addition to reductions in the entry number of zone A, zone B, and zone C by 2.77-fold, 7.92-fold, and 4.18-fold, respectively, relative to the control group. In contrast, co-treatment with DAPA led to a 5.25-fold increase in the total distance traveled along with a rise in the number of entries into zone A, zone B, and zone C by 3.18-fold, 8.25-fold, and 4.12-fold, respectively, relative to the DOX group, as shown in Fig. 4, indicating that DAPA improved learning, working memory, and reference memory.

### Impact of Dapagliflozin on Forced Swimming Test (FST)

**Fig. 5:**
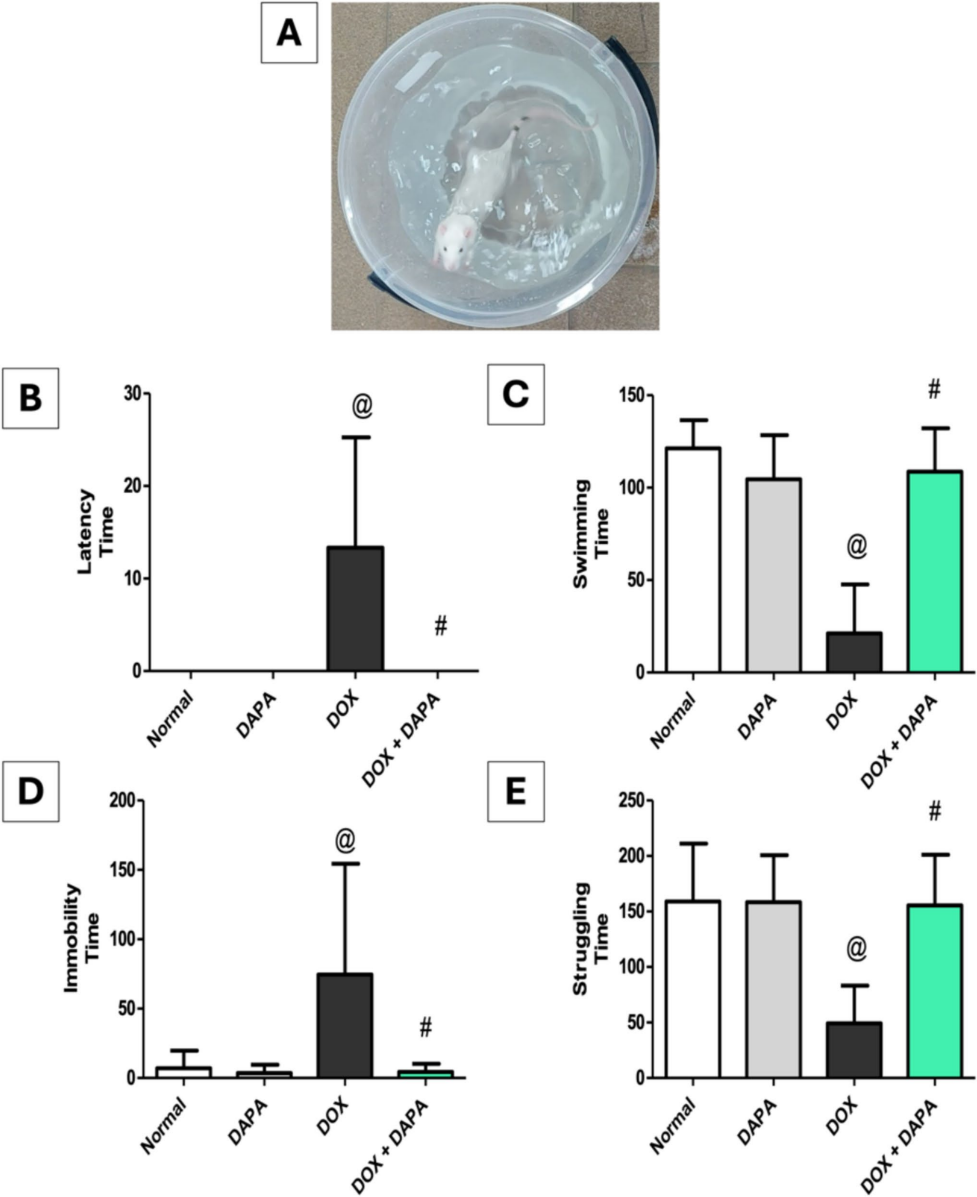
Effect of dapagliflozin on forced swimming test (FST) against doxorubicin-induced chemobrain in rats. **A** rats on forced swimming test apparatus, **B** latency time, **C** swimming time, **D** immobility time, and **E** struggling time. *DAPA* Dapagliflozin, *DOX* Doxorubicin. **@:** vs. the control group & **#:** vs. DOX-intoxicated group, P < 0.05

DOX rats exhibited depression-like behavior in FST, manifested by a decrease in swimming time and struggling time by 5.73-fold and 3.22-fold, respectively, with a prolonged immobility time of 10.39-fold relative to the control group. Besides, the DOX animals recorded the highest values of latency time. Inversely, DAPA-cotreated rats exhibited prolonged swimming time and struggling time by 5.13-fold and 31.15-fold, respectively, with a reduction in immobility time by 17.21-fold in contrast to the DOX group. Likewise, the animals recorded low latency time values, indicating that DAPA treatment can attenuate the depression-like behavior induced by DOX (Fig. 5).

### Histopathology Findings

**Fig. 6:**
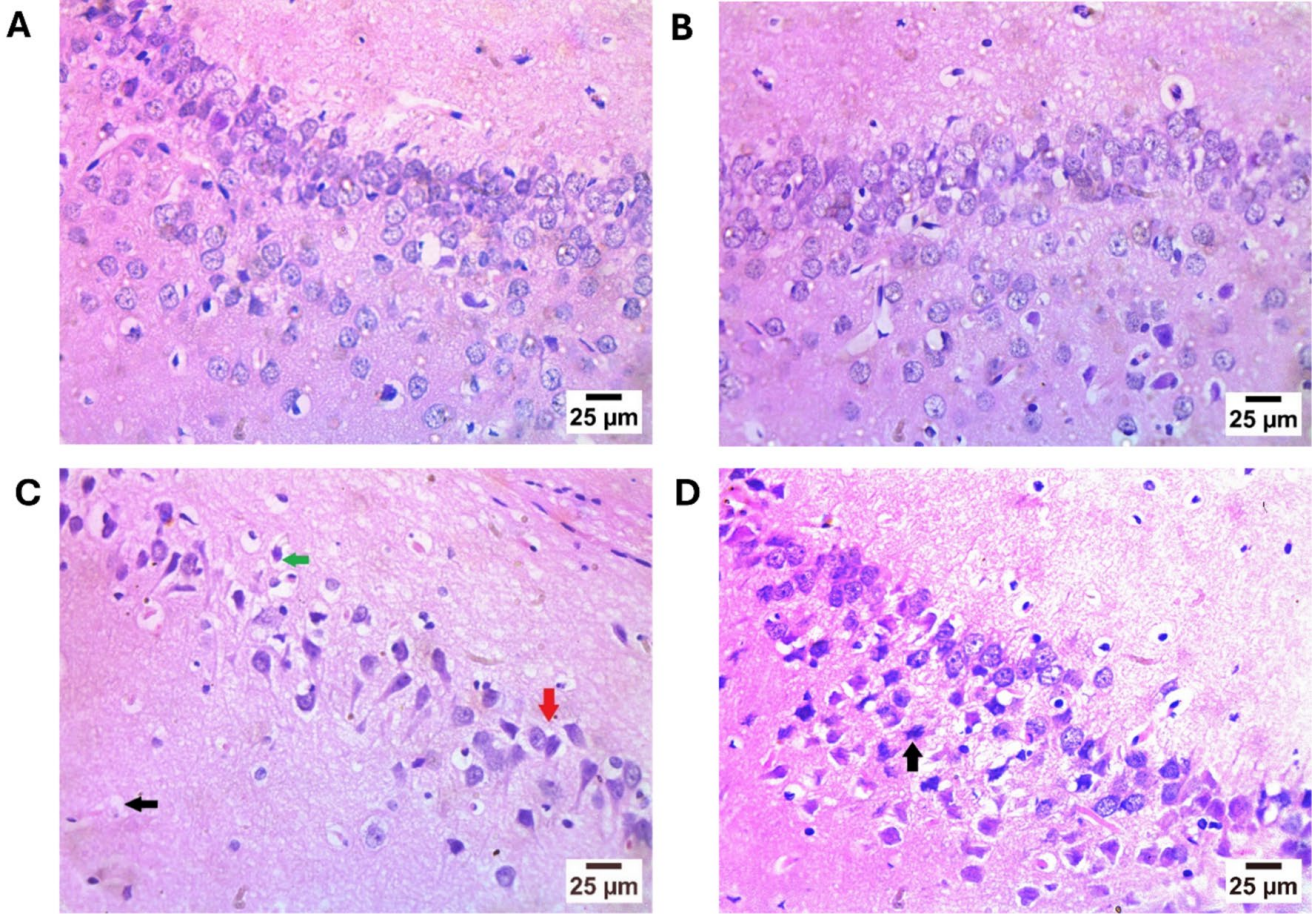
Representative H&E histopathological photomicrographs. The control group **(A)** and DAPA group **(B)** revealed normal histological structure in the CA1 zone of the hippocampus. In contrast, DOX-intoxicated group **(C)** resulted in the disorganization of pyramidal cells (black arrow) and the presence of degeneration and shrinkage of some pyramidal cells (green arrow) in the CA1 zone of the hippocampus. Whereas the photomicrograph of DOX + DAPA treatment **(D)** showed degeneration in a few numbers of the pyramidal cells (black arrow) in the CA1 zone of the hippocampus. *CA1* hippocampal cornu ammonis neurons, *DAPA* Dapagliflozin, *DOX* Doxorubicin, and *H&E* Hematoxylin and Eosin stain

In Fig. 6, the histopathology analysis revealed that the control and DAPA groups’ hippocampal CA1 histological structures were normal. The photomicrographs of the DOX group showed the presence of disorganized pyramidal cells, in addition to some pyramidal cells degenerating and shrinking with a little vacuolation in the hippocampal CA1 zone’s neuropil of the molecular layer. In contrast, the photomicrographs of the DOX + DAPA group demonstrated the presence of degeneration in a few numbers of the pyramidal cells in the hippocampal CA1 zone.

### Assessment of Total Caspase-3 and NF-κB-p65 by Immunohistochemistry

**Fig. 7:**
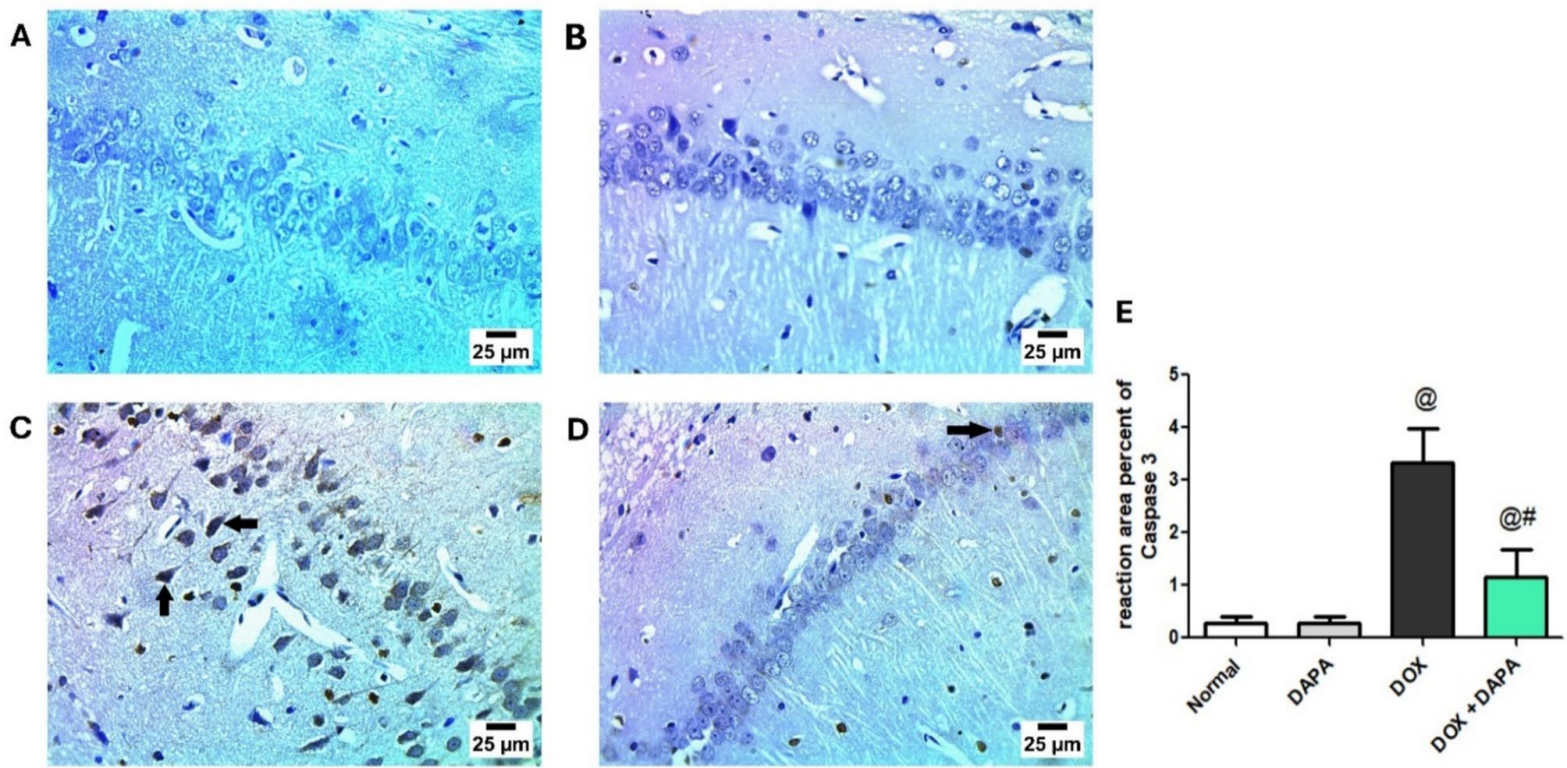
Representative immunohistochemistry photomicrographs of total caspase-3. The control group **(A)** and DAPA group **(B)** showed negative reactions for caspase-3 in the hippocampus’s CA1 area. In contrast, DOX-intoxicated group **(C)** resulted in a positive reaction for caspase-3 in pyramidal cell nuclei (arrows) in the hippocampus’s CA1 area. The photomicrograph of DOX + DAPA treatment **(D)** showed a mild positive reaction for caspase-3 in a few numbers of pyramidal cell nuclei (arrow) in the hippocampal CA1 area. Panel **(E)** Reaction area percent of Caspase-3. *CA1* hippocampal cornu ammonis neurons, *DAPA* Dapagliflozin, and *DOX* Doxorubicin. **@:** vs. the control group & **#:** vs. DOX-intoxicated group, P < 0.05

Based on the immunohistochemistry outcomes regarding caspase-3 (Fig. 7), DOX showed a positive reaction for caspase-3 in the cytoplasm and nucleus of the pyramidal cells (Fig. 7C), with an increased reaction area % by 11.86-fold relative to the control group (P < 0.001) (Fig. 7E). In contrast, DAPA co-treatment showed a mild positive reaction for caspase-3 in a limited number of cytoplasm and nuclei of pyramidal cells in the CA1 region of the hippocampus (Fig. 7D), with a 2.91*-*fold increase in the reaction area % relative to the DOX group (Fig. 7E).

**Fig. 8:**
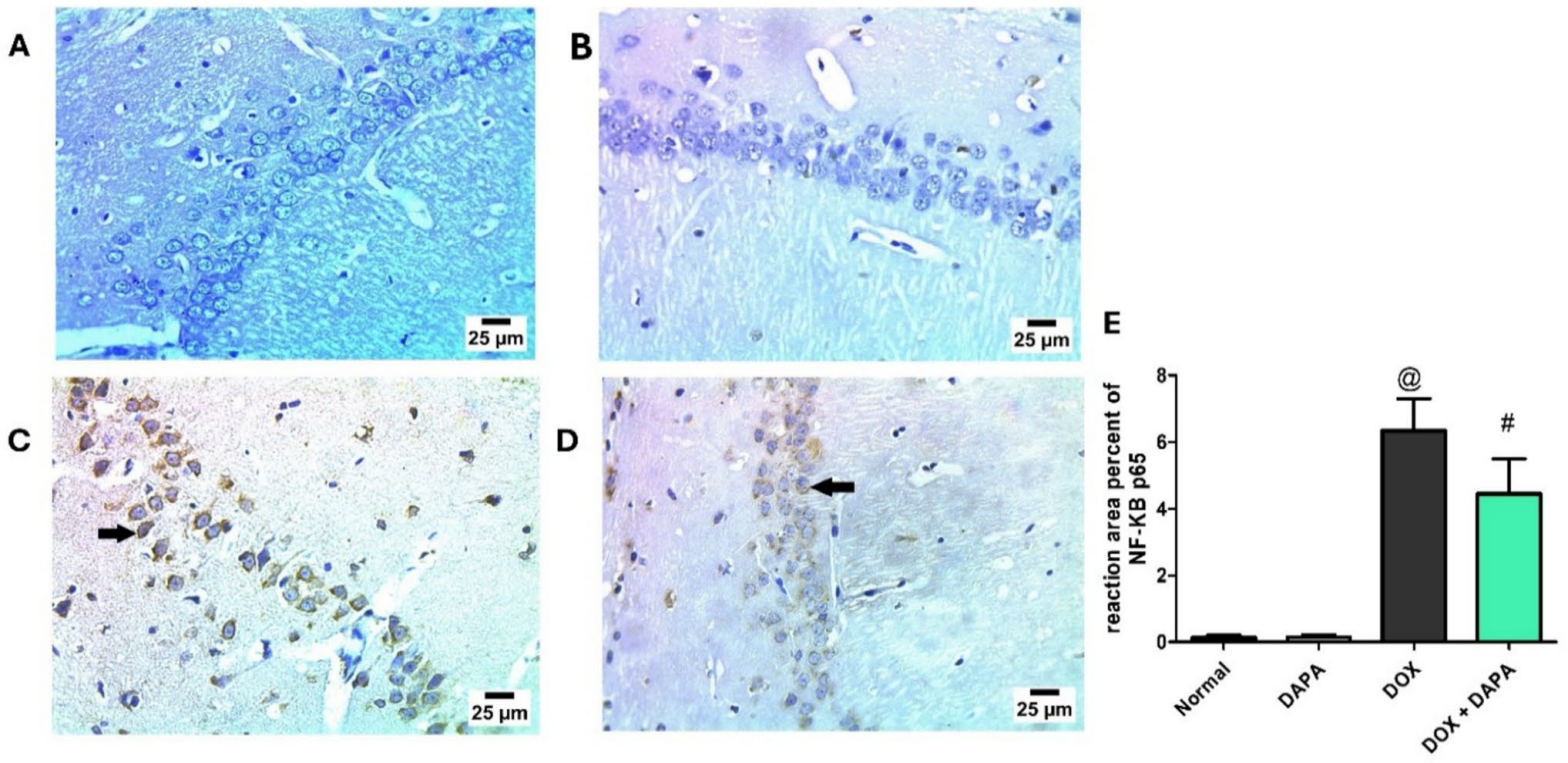
Representative immunohistochemistry photomicrographs of NF-κB-p65. Control groups **(A)** and DAPA group **(B)** showed negative reactions for NF-κB-p65 in the hippocampus’s CA1 area. In contrast, DOX administration **(C)** resulted in a positive reaction for NF-κB-p65 in the neuron cytoplasm of pyramidal layers (arrows) in the hippocampus’s CA1 area. The photomicrograph of DOX + DAPA treatment **(D)** demonstrated a mild positive reaction for NF-κB-p65 in the neuron cytoplasm of pyramidal layers (arrow) in the hippocampus’s CA1 area. Panel **(E)** Reaction area percent of NF-κB-p65. *CA1* hippocampal cornu ammonis neurons, *DAPA* Dapagliflozin, *DOX* Doxorubicin, and *NF-κB-p65* Nuclear factor-kappa B-p65. **@:** vs. the control group & **#:** vs. DOX-intoxicated group, P < 0.05

Figure 8 revealed marked increases in NF-κB p65 expression in both the cytoplasm and nucleus of pyramidal cells following DOX administration (Fig. 8C), demonstrating a 48.85-fold elevation in the reaction area % compared to the control group (P < 0.001) (Fig. 8E). Conversely, co-treatment with DAPA resulted in a mild NF-κB p65 immunoreactivity, which was observed in a limited number of cytoplasmic and nuclear compartments of pyramidal cells within the CA1 region of the hippocampus **(**Fig. 8D**)**, corresponding to a 1.43-fold increase in the reaction area % relative to the DOX-treated group (Fig. 8E).

### Impact of Dapagliflozin on Oxidative Stress Markers in Brain Tissue Homogenates

**Fig. 9:**
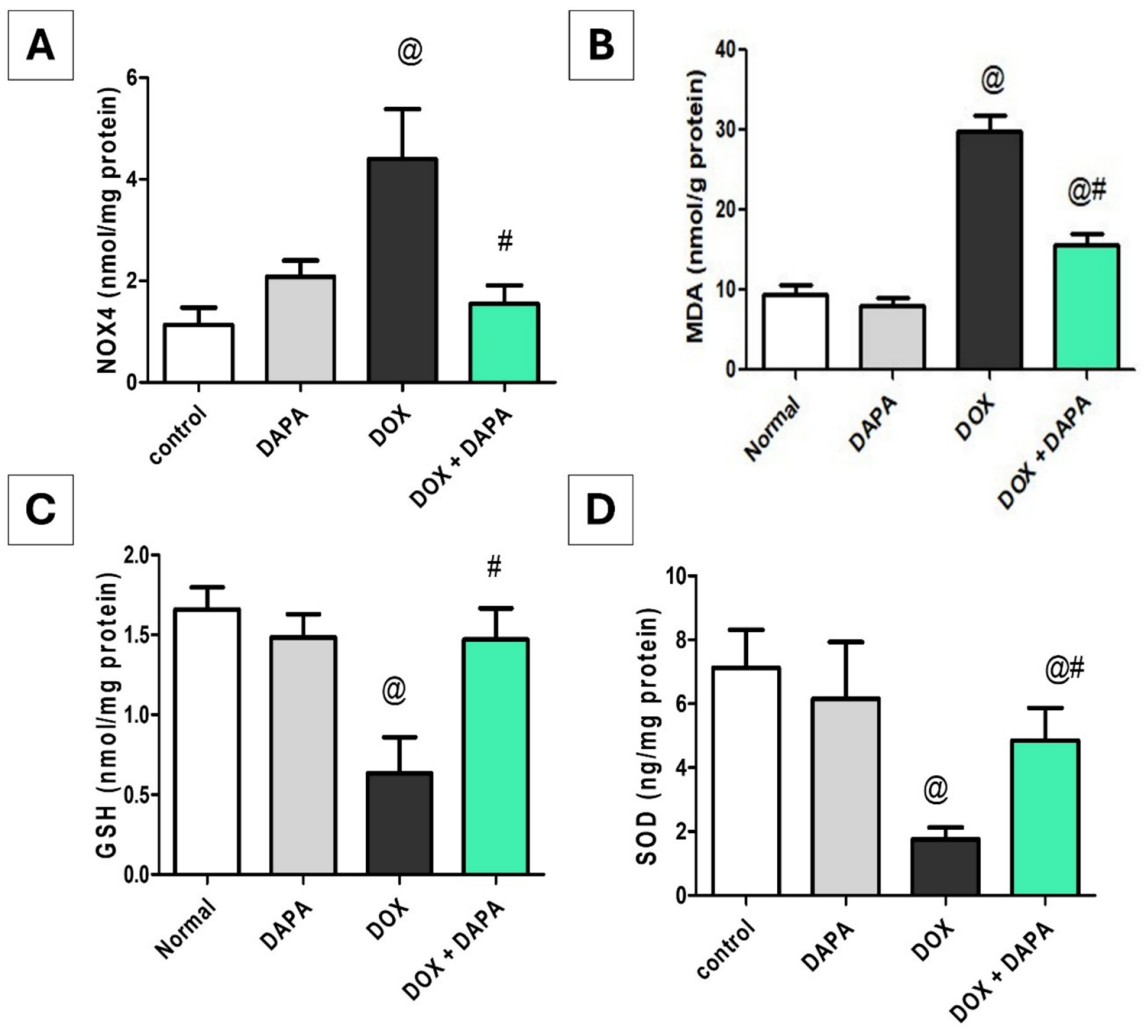
Effect of dapagliflozin on oxidative stress biomarkers against doxorubicin-induced chemobrain in rats. **A** brain tissue content of NOX4, **B** brain tissue content of MDA, **C** brain tissue content of GSH, and **D** brain tissue content of SOD. *DAPA* Dapagliflozin, *DOX* Doxorubicin, *GSH* Reduced glutathione, *MDA* Malondialdehyde, *NOX4* NADPH oxidase 4 and *SOD1* Superoxide dismutase 1. **@:** vs. the control group & **#:** vs. DOX-intoxicated group, P < 0.05

DOX-induced oxidative stress was manifested by a significant decrease in the brain antioxidant tissue content (GSH; 2.63-fold, P < 0.001) and (SOD; 4.07-fold, P < 0.001). Consequently, DOX administration revealed significant increases in NOX4 (3.89-fold, P < 0.001) and MDA (3.15-fold, P < 0.001) relative to the control group. Whereas the co-treatment of DAPA notably increased GSH by 2.33-fold (P < 0.001) and SOD by 2.77-fold (P < 0.001) in addition to a marked decrease in NOX4 and MDA by 2.84-fold and 1.91-fold, respectively (P < 0.001), in comparison to the DOX group (Fig. 9).

### Impact of Dapagliflozin on Inflammatory Markers in Brain Tissue Homogenates

**Fig. 10:**
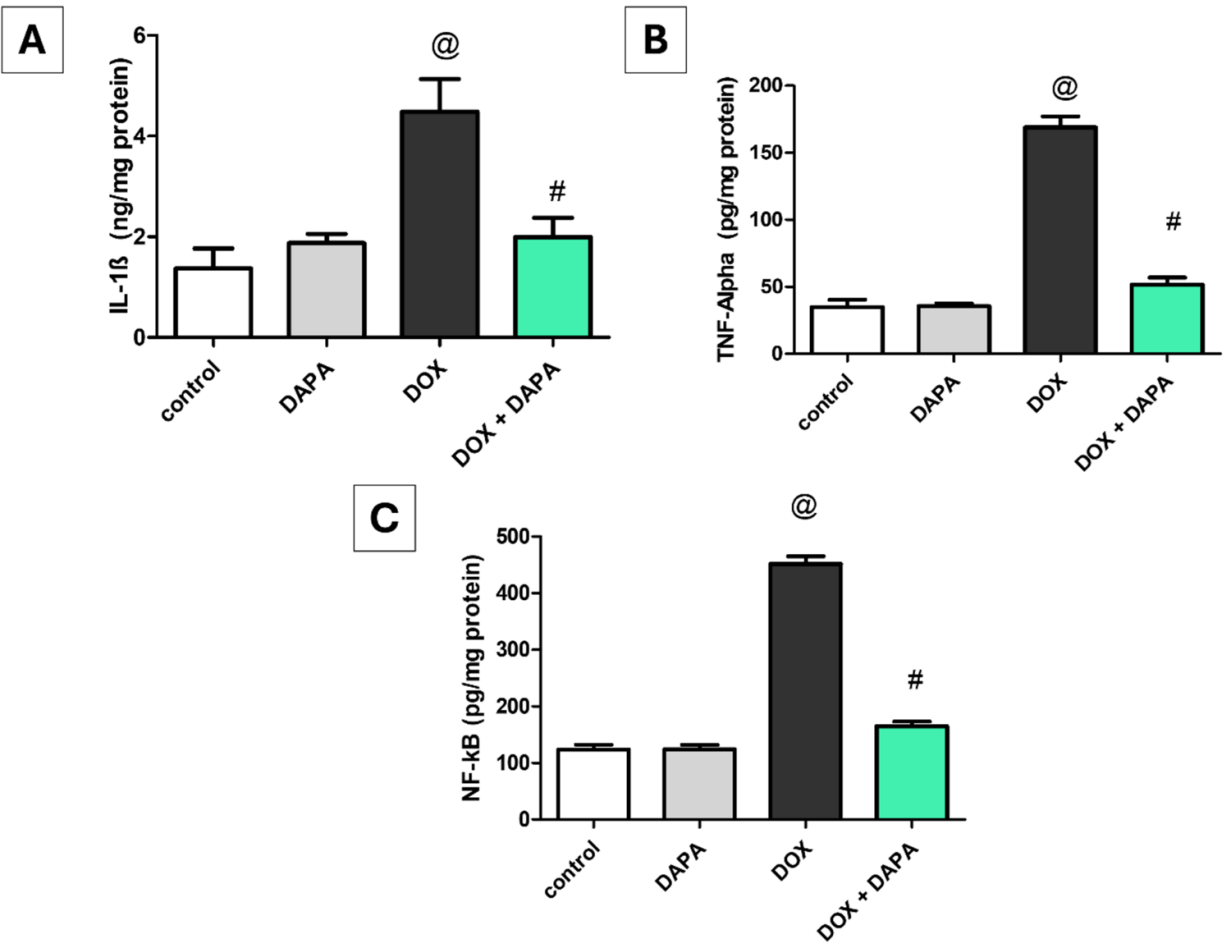
Effect of dapagliflozin on inflammatory markers against doxorubicin-induced chemobrain in rats.** A** brain tissue content of IL-1β, **B** brain tissue content of TNF-α, and **C** brain tissue content of NF-κB. *DAPA* Dapagliflozin, *DOX* Doxorubicin, *IL-1β* Interleukin-1β, *NF-κB* Nuclear factor-kappa B and *TNF-α* Tumor necrosis factor-alpha. **@:** vs. the control group & **#:** vs. DOX-intoxicated group, P < 0.05

DOX-induced neuroinflammation was manifested by significant increases in the brain tissue levels of IL-1β (277.01%), TNF-α (384.65%), and NF-κB (265.29%) relative to the control group (P < 0.001). Whereas, in comparison to the DOX group, DAPA co-treatment reduced the brain tissue levels of IL-1β (2.25-fold, P < 0.001), TNF-α (3.27-fold, P < 0.001), and NF-κB (2.74-fold, P < 0.001), respectively (Fig. 10).

### Impact of Dapagliflozin on AKT/GSK-3β and Wnt/β-Catenin Signaling Cascades

**Fig. 11:**
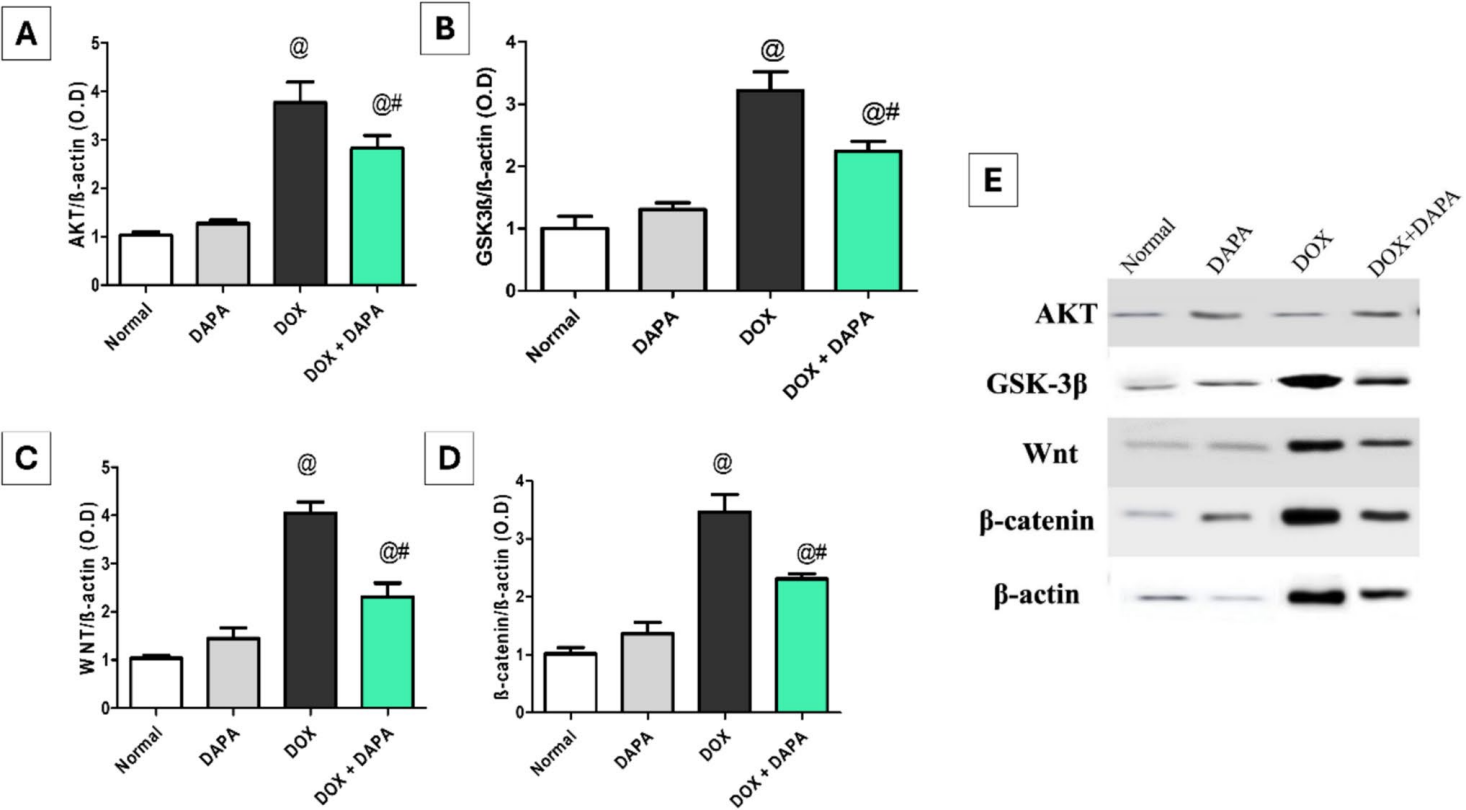
Effect of dapagliflozin on AKT/GSK-3β and Wnt/β-catenin signaling cascades against doxorubicin-induced chemobrain in rats.** A** brain tissue content of AKT, **B** brain tissue content of GSK-3β**, C** brain tissue content of Wnt**, D** brain tissue content of β-catenin, and **E** western blots. *AKT* Protein kinase B, *DAPA* Dapagliflozin, *DOX* Doxorubicin, *GSK-3β* Glycogen synthase kinase-3beta, *Wnt* Wingless-related integration site, and *β-Catenin* beta-Catenin. **@:** vs. the control group & **#:** vs. DOX-intoxicated group, P < 0.05

DOX administration induced a marked elevation in the protein levels of brain AKT and GSK-3β by 3.42-fold (P < 0.001) and 3.22-fold (P < 0.001), respectively, in contrast to the control group. Likewise, Wnt and β-catenin contents were also increased in the DOX group by 3.88-fold (P < 0.001) and 3.66-fold (P < 0.001), respectively, in comparison with the control group. On the contrary, DAPA co-treatment effectively suppressed the protein levels of AKT, GSK-3β, Wnt, and β-catenin by 24.93%, 30.43%, 42.82%, and 33.43%, respectively (P < 0.001), in contrast to the DOX group (Fig. 11).

## Discussion

Many cancer patients and survivors encounter cognitive impairments during and after chemotherapy, sometimes termed “chemobrain” or “chemofog”. The patients experience deficits in executive function, attention, and memory [27]. Although doxorubicin is extensively used in chemotherapy, its application is constrained because of the significant side effects it induces. DOX-induced cognitive impairment has garnered significant interest due to its detrimental impact on the quality of life of cancer survivors [28].

Recent preclinical trials have revealed that dapagliflozin may have protective effects on neural tissue by lowering oxidative stress, neuroinflammation, and apoptosis [11, 29]. Preclinical animal studies showed that dapagliflozin could improve cognitive problems caused by neurotoxins by lowering the generation of ROS and decreasing microglia-mediated inflammatory responses [30]. We aimed in this study to investigate DAPA effect on DOX-induced chemobrain and to investigate its ability to ameliorate DOX-induced disruption in memory function.

The current study revealed that systemic injection of DOX resulted in cognitive decline, as mentioned previously [31–33]. The cognitive deterioration generated by DOX was evidenced by impaired spatial memory in the NORT, where DOX rats failed to differentiate between familiar and novel objects relative to healthy controls. Furthermore, animals treated with DOX demonstrated a decline in locomotor activity and exploratory behavior in the OFT, evidenced by reductions in distance traveled, number of corner zone entries, mean speed, and number of line crossings. Moreover, DOX impairs the reference memory of rats, resulting in diminished exploration of novel environments, as indicated by significant declines in both the total distance traveled and the frequency of entries into zones A, B, and C in the Y-maze apparatus. The outcomes of the FST revealed that DOX rats exhibited depressive-like behavior, characterized by reduced swimming and struggle duration, alongside elevated latency and immobility time. The deleterious impact of DOX on behavioral outcomes was corroborated histologically by the disorganization, degeneration, and atrophy of pyramidal cells, accompanied by minor vacuolation in the neuropil of the hippocampal molecular layer.

Conversely, DAPA-cotreatment showed markedly enhanced recognition memory in the NORT, as indicated by prolonged exploration time for both familiar and novel items, alongside an increase in the number of entries to both, as previously reported [34, 35]. Rats co-treated with DAPA exhibited greater exploratory and locomotor activity in the open field test, evidenced by significant elevations in distance traveled, corner zone entry, mean speed, and line crossings, corroborating prior research [12, 35, 36]. Furthermore, DAPA therapy enhanced learning, working memory, and reference memory, as evidenced by increased total distance traveled in the Y-maze and elevated entries in zones A, B, and C, supporting earlier studies [34]. The duration of swimming and struggling in FST was increased following treatment with DAPA, accompanied by a significant reduction in latency and immobility time, indicating that DAPA may exhibit antidepressant behavior, in accordance with prior studies [37, 38]. Histologically, the DAPA co-treatment group exhibited reduced degeneration of pyramidal cells in the CA1 region of the hippocampus relative to the DOX group. Taken together, all the previously mentioned outcomes substantiate the efficacy of DAPA treatment in attenuating the neuropsychological impact of DOX-induced chemobrain and reinstating the brain’s normal function and structure.

The molecular pathogenesis of DOX-induced chemobrain is attributed to excessive generation of ROS or the decline in the antioxidant defense, resulting in oxidative stress that has been linked to neuroinflammation and neurotransmitter imbalance [39, 40]. DOX triggers the overproduction of ROS through activation of enzymes such as NOX4, which mediates mitochondrial superoxide generation, leading to mitochondrial dysfunction and oxidative damage. This ROS overproduction initiates a cascade of cellular events culminating in oxidative damage to proteins, lipids, and DNA [41]. Even though DOX cannot cross the blood–brain barrier (BBB), it can cause substantial neurotoxicity with neuronal and synaptic loss, prompting cognitive deficits in the brain [39, 42] In the current work, DOX-induced oxidative stress was proved by higher levels of NOX4 and MDA, accompanied by loss of antioxidant defense mechanisms shown as lower GSH and SOD activities. These findings are in accordance with previously published studies [43, 44]. Endogenous NOX4 is essential for mediating mitochondrial superoxide production, resulting in mitochondrial dysfunction and cell apoptosis [45]. MDA is a byproduct of ROS—peroxidation of polyunsaturated fatty acids [46]. SOD is an antioxidant enzyme that transforms superoxide radicals into hydrogen and oxygen, which is a key step in protecting cells from oxidative stress [47]*.* Also, the GSH is important for detoxification pathways and the possible maintenance of redox reaction pathways [48]. On the other hand, DAPA mitigated DOX—evoked oxidative stress by reducing the tissue contents of NOX4 and MDA while simultaneously elevating the production of GSH and SOD, which are key antioxidant molecules, consistent with earlier findings [49, 50]. DAPA has been confirmed to mitigate oxidative stress by inhibiting the formation of free radicals [51]. Depending on these findings, the antioxidant effect of DAPA contributes to the alleviation of DOX-induced chemobrain.

Notably, in the present study, DOX increased the production of inflammatory markers, including IL-1β, TNF-alpha, and NF-κB. This elevation in cytokine levels was linked to neural inflammation and subsequent memory impairments. These findings are consistent with prior published investigations [33, 52, 53]. IL-1β binds to IL-1R1 (present in neurons, astrocytes, and microglia in the central nervous system) and initiates intracellular signaling pathways that activate downstream signaling pathways through NF-κB and mitogen-activated protein kinase (MAPK) and subsequently lead to the production of pro-inflammatory cytokines, including TNF-α, IL-6, and chemokines [54]. Moreover, TNF-α is crucial in brain inflammation through the induction of adhesion molecules on endothelial cells to facilitate the influx of peripheral immune cells into the CNS and enhance the inflammatory response [55]. In the same context, our immunohistochemistry outcomes confirmed the neuroinflammatory effect of DOX through a positive reaction for NF-KB-p65 in the neurons cytoplasm of pyramidal layers of the hippocampus. SGLT-2 inhibitors are recognized for their ability to reduce the production of pro-inflammatory cytokines, thereby suppressing the inflammatory response [42]. Hence, our results indicate that DAPA decreased DOX—related inflammatory markers, such as IL-1β, TNF-α, and NF-ĸB production, which is consistent with previously published studies [42, 51, 56, 57]. These findings emphasize the neuroprotective effects of DAPA as a consequence of its antioxidant and anti-inflammatory capabilities, which prevent DOX—induced cognitive impairment in the brain.

In addition to oxidative stress and the resulting inflammatory responses that significantly exacerbate DOX—induced neurotoxicity, DOX induces apoptotic cell death in neuronal tissues by modulating key apoptotic signaling pathways. It alters the balance of B-cell lymphoma 2 (Bcl-2) family proteins, favoring pro-apoptotic members that promote mitochondrial outer membrane permeabilization and the release of cytochrome c. This activates downstream caspases such as caspase-3 and caspase-9, executing programmed cell death. Furthermore, DOX triggers p53-dependent apoptotic pathways that potentiate neuronal apoptosis within the brain’s neurogenic regions and exacerbate cognitive impairments [41, 58]. The current study demonstrated that neuronal apoptosis induced by DOX was evidenced by a positive immunohistochemical reaction for caspase-3 and NF-κB in the hippocampus, alongside the stimulation of protein kinase B (AKT), glycogen synthase kinase-3beta (GSK-3β), wingless-related integration site (Wnt), and beta-catenin (β-catenin) apoptotic signaling proteins, which contribute to cognitive impairment; these findings are consistent with previously published research [7, 59, 60]. Caspase-3 is implicated in synaptic degeneration and precipitates neuronal death [61]. AKT hyperactivation induces oxidative damage in neural tissue, expediting neuronal death and damaging brain cells [62, 63]*.* Furthermore, elevated GSK-3β activity has been related to neuronal death, particularly in the hippocampus and cortex [64]. Overexpression of AKT/GSK-3β reduces mitochondrial ATP output, increases oxidative stress, and exacerbates apoptotic effects by activating caspase-3 and releasing cytochrome C [13]. Likewise, the AKT/GSK-3β signaling pathway contributes to DOX-induced memory deficits by modulating NF-ĸB signal transduction, leading to the stimulation of apoptotic pathways in the brain [13, 32, 65–67]. Wnt signaling is triggered by DOX-induced oxidative imbalance and DNA degradation, resulting in the stabilization and translocation of β-catenin. This activation also promotes apoptosis by upregulating pro-apoptotic genes such as Bcl-2-associated X protein (Bax) and p53, while downregulating anti-apoptotic genes like Bcl-2 [68].

On the contrary, DAPA protected neurons from DOX-induced apoptosis, as revealed immunohistochemically by a mild positive reaction for caspase-3 and NF-κB. DAPA also inhibited the downstream AKT/GSK-3β and Wnt/β-catenin pathways, reducing brain inflammation and apoptosis. These findings were consistent with a previously published study on DAPA, which displays its anti-apoptotic properties by reducing macrophage polarization through attenuation of the phosphoinositide 3-kinase (PI3K)/AKT signaling pathway [69]. In the same context, Xuan et al. (2022) found that DAPA provides renoprotective effects through reducing the Wnt3α/β-catenin/GSK-3β signaling pathway, alleviating renal fibrosis in Sprague–Dawley rats [70]. Collectively, DAPA mitigates DOX-induced activation of AKT/GSK-3β and Wnt/β-catenin apoptotic pathways, leading to reduced oxidative stress, neuroinflammation, and neuronal death. This, in turn, improves the detrimental effects of DOX on brain function and structure.

A limitation of the present study is the exclusive use of male rats, which may limit the generalizability of the findings across sexes. This population restriction may overlook potential sex-related variations in response to DAPA treatment. Accordingly, future studies should consider including female animal models to investigate possible sex differences in therapeutic response. Additionally, long-term assessments of cognitive function following DAPA administration are essential to evaluate the persistence and safety of its effects. These future investigations will contribute to a more comprehensive understanding of DAPA’s efficacy and underlying mechanisms in diverse biological contexts.

## Conclusion

Our findings demonstrate that DAPA significantly reduced DOX-induced chemobrain in rats by regulating major oxidative stress indicators. DAPA specifically decreased NOX4 and MDA brain tissue content while increasing antioxidant defenses through the induction of SOD and GSH. DAPA reduced inflammatory biomarkers such as TNF-α, IL-1β, and NF-ĸB, counteracting the inflammatory sequences of DOX. Furthermore, DAPA reduced apoptosis via suppression of the AKT/GSK-3β and Wnt/β-catenin signaling cascades. These findings were supported using cognitive behavioral tests and biochemical tests, as well as immunohistopathological investigations of caspase-3 and NF-κB. The findings of this study demonstrate that DAPA has the potential to be repurposed to assist individuals with memory impairment who are undergoing chemotherapy with DOX. This underscores the necessity of conducting clinical trials.

## Supplementary Information

Below is the link to the electronic supplementary material.

Supplementary file1 (PDF 624 KB)

## Data Availability

All data are available on reasonable request with the corresponding author.
