# Supplementary material for: Dapagliflozin Ameliorates Doxorubicin-Induced Chemobrain and Cognitive Abnormalities in Rats: Modulation of AKT/GSK-3β and Wnt/β-Catenin Pathways
Source: Neurochem Res. 2025 Sep 5;50(5):286. doi: 10.1007/s11064-025-04538-0 (PMC12413430; doi:10.1007/s11064-025-04538-0)
Supplement: Supplementary file 1 — Supplementary file1 (PDF 624 KB) [file 11064_2025_4538_MOESM1_ESM.pdf]

Tel:240-252-7368(USA)
Fax:240-252-7376(USA)
www.elabscience.com
E-mail:techsupport@elabscience.com
Elabscience Biotechnology Inc.

Elabscience®

# Caspase-3 Polyclonal Antibody

| | | | |
|---|---|---|---|
| **Catalog No.** | E-AB-63602 | **Reactivity** | H,M,R |
| **Storage** | Store at -20°C. Avoid freeze / thaw cycles. | **Host** | Rabbit |
| **Applications** | IHC,IF | **Isotype** | IgG |

**Note:** Centrifuge before opening to ensure complete recovery of vial contents.

## Images

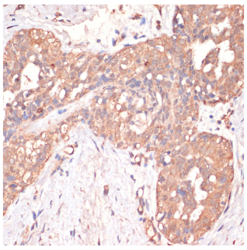

Immunohistochemistry of paraffin-embedded Human mammary cancer using Caspase-3 Polyclonal Antibody at dilution of 1:200 (40x lens).

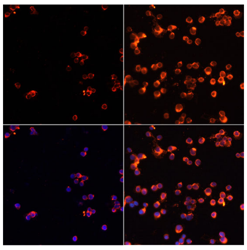

Immunofluorescence analysis of Jurkat cells using Caspase-3 Polyclonal Antibody at dilution of 1:100. Jurkat cells treated by Etoposide 25uM etoposide for 5 hours (left). Blue: DAPI for nuclear staining.

## Immunogen Information

| | |
|---|---|
| **Immunogen** | Recombinant fusion protein of human Caspase-3 (NP_004337.2). |
| **GeneID** | 836 |
| **Swissprot** | P42574 |
| **Synonyms** | CPP32,CPP32B,SCA-1,Active Caspase 3,CASP3,active Caspase-3,Caspase-3 p12 |

## Product Information

| | |
|---|---|
| **Buffer** | PBS with 0.02% sodium azide, 50% glycerol, pH7.3. |
| **Purify** | Affinity purification |
| **Dilution** | IHC 1:50-1:100 IF 1:50-1:100 |

## Background

This gene encodes a protein which is a member of the cysteine-aspartic acid protease (caspase) family. Sequential activation of caspases plays a central role in the execution-phase of cell apoptosis. Caspases exist as inactive proenzymes which undergo proteolytic processing at conserved aspartic residues to produce two subunits, large and small, that dimerize to form the active enzyme. This protein cleaves and activates caspases 6, 7 and 9, and the protein itself is processed by caspases 8, 9 and 10. It is the predominant caspase involved in the cleavage of amyloid-beta 4A precursor protein, which is associated with neuronal death in Alzheimer's disease. Alternative splicing of this gene results in two transcript variants that encode the same protein.

For Research Use Only
Thank you for your recent purchase.
If you would like to learn more about antibodies,please visit www.elabscience.com.
Focus on your research
Service for life science

Applications:WB-Western Blot IHC-Immunohistochemistry IF-Immunofluorescence IP-Immunoprecipitation FC-Flow cytometry ChIP-Chromatin Immunoprecipitation Reactivity: H-Human R-Rat M-Mouse Mk-Monkey Dg-Dog Ch-Chicken Hm-Hamster Rb-Rabbit Sh-Sheep Pg-Pig Z-Zebrafish X-Xenopus C-Cow.
